# Modeling of Slot Waveguide Sensors Based on Polymeric Materials

**DOI:** 10.3390/s110807327

**Published:** 2011-07-25

**Authors:** Paolo Bettotti, Alessandro Pitanti, Eveline Rigo, Francesco De Leonardis, Vittorio M. N. Passaro, Lorenzo Pavesi

**Affiliations:** 1 Nanoscience Laboratory, Department of Physics, University of Trento, via Sommarive 14, 38123 Povo, Italy; E-Mails: alessandro.pitanti@sns.it (A.P.); rigo@science.unitn.it (E.R.); pavesi@science.unitn.it (L.P.); 2 NEST, Scuola Normale Superiore and Istituto Nanoscienze-CNR, piazza San Silvestro 12, 56127 Pisa, Italy; 3 Department of Physics, University of Modena and Reggio Emilia, via Campi 213/A, 41100 Modena, Italy; 4 Dipartimento di Ingegneria dell’Ambiente e per lo Sviluppo Sostenibile, Politecnico di Bari, Viale del Turismo 8, 74100 Taranto, Italy; E-Mail: f.deleonardis@poliba.it; 5 Dipartimento di Elettrotecnica ed Elettronica, Politecnico di Bari, Via E. Orabona 4, 70125 Bari, Italy; E-Mail: passaro@deemail.poliba.it

**Keywords:** slot waveguide, polymeric materials, optical sensor, 07.07.Df Sensors, 42.70.Jk Polymers and organics, 42.79.Gn Optical waveguides and couplers

## Abstract

Slot waveguides are very promising for optical sensing applications because of their peculiar spatial mode profile. In this paper we have carried out a detailed analysis of mode confinement properties in slot waveguides realized in very low refractive index materials. We show that the sensitivity of a slot waveguide is not directly related to the refractive index contrast of high and low materials forming the waveguide. Thus, a careful design of the structures allows the realization of high sensitivity devices even in very low refractive index materials (e.g., polymers) to be achieved. Advantages of low index dielectrics in terms of cost, functionalization and ease of fabrication are discussed while keeping both CMOS compatibility and integrable design schemes. Finally, applications of low index slot waveguides as substitute of bulky fiber capillary sensors or in ring resonator architectures are addressed. Theoretical results of this work are relevant to well established polymer technologies.

## 1. Introduction

Nowadays, optical sensing is a very promising and powerful technology, which is finding widespread ranges of applications, such as environment, security, health. For example, the increased demand of *in situ* diagnostic tools requires the development of small, reliable and cheap sensing devices. Nowadays, high refractive index (HI) group IV semiconductor materials, such as silicon on insulator, play a major role in the development of integrated optical sensors. This is mainly due to the possibility to have strong optical field confinement and, then, extremely small footprint devices. On the other hand, the silicon transparency window is limited to the range 1–8 μm (with a propagation loss less than 2 dB/cm). Moreover, the use of low refractive index (LI) materials (polymers) for realizing high sensitivity integrated optical sensors should have a number of advantages compared to high index (HI) dielectrics, especially:

- LI sensors can exploit the polymer transparency in the visible range, extending the usual working window of integrated sensors and enabling the fabrication of simpler and cheaper devices;
- LI materials have a much greater chemical flexibility than HI semiconductor materials. In fact, it is possible to design polymer synthetic materials with *ad-hoc* functionalities. Polymers can be surface functionalized with a number of different chemicals using a number of fabrication methods for waveguide structuring. Moreover, they can be doped with optically active centres (such as dye molecules, quantum dots), realizing active waveguides (WG) and WG arrays with tailored optical properties that can be optically pumped with simple LEDs;
- LI material fabrication is much simpler than using HI dielectrics, and greater structural quality is expected. A single lithographic/imprinting step is enough to create the waveguide without any dry etching step. Thus, small roughness is expected;
- LI materials are environmentally more friendly and require smaller efforts in their disposal.

It is generally believed that LI materials cannot rival with the performance of HI materials in terms of sensitivity, especially in integrated optics. In this paper we theoretically demonstrate that LI dielectrics can reach comparable performances with HI materials in properly designed photonic structures (namely slot waveguides, SWGs) and that the sensitivity of LI SWG is slightly reduced by a factor much smaller than the ratio of the material refractive indices forming the waveguide. Thus, we propose the use of vertical LI SWGs as WG for chemical sensors and compare them to analogous WG fabricated in HI materials. We also describe the behaviour of SWG with multiple slots and propose some applications of such SWGs in absorption/fluorescent sensing devices.

## 2. Integrated Optical Sensing by Evanescent Field

### 2.1. Principles of Operation

Optical sensing in waveguide-based structures requires the interaction between the guided optical mode and the environment. Sensor response is usually proportional to the field intensity available for this interaction. Usually, the interaction can be achieved by using evanescent fields (such as in usual strip WG geometry [1]) or surface waves (as in plasmons [2]). A strong field confinement is commonly considered as a benefit in integrated optics, but we can remember that sensitivity (***S***) scales inversely proportional to the optical mode confinement in the waveguide [3]. Thus, it is usually needed to find a trade-off between the power confined inside the WG core and ***S***. These two parameters are inversely correlated with the mode confinement: while the former is proportional to it, the latter is maximized for less confined modes.

### 2.2. Slot Waveguide Approach

Slot Waveguides (SWG) are becoming well-known guiding photonic structures, although recently proposed [4]. For quasi-TE polarization, a slot WG shows a peculiar field concentration mechanism in the low refractive index region. This is due to the discontinuity of the field components normal to the dielectric interfaces. This guiding structure is particularly suitable for optical sensing because of the large mode confinement factor (CF) in the slot region. A SWG can be realized in two geometrical configurations that differ by the orientation of the low index region, either vertically etched at a rectangular WG centre or horizontally sandwiched between two high index layers. These different geometrical orientations have important implications on the fabrication process and relevant tolerances (particularly if dry etching steps are needed). In particular, the horizontal scheme can be fabricated by thin film deposition technologies and, thus, nanometre resolution on slot height is easily achieved together with very small roughness [5]. Despite some recent works demonstrating the possibility to use horizontal SWGs as chemical sensors [6,7], their use is limited up to now to gas sensing measurements because of the difficulty to fill them with liquids. On the contrary, vertical SWGs can be easily infiltrated using standard laboratory equipment, such as a micropipette dispenser coupled to an optical microscope. On the other hand, vertical SWG requires both high resolution lithography and etching processes, which can degrade the surface quality. In fact, in vertical SWGs losses strongly depend on surface imperfections and reach very high values in HI materials, even if state of the art technologies are employed during fabrication [8,9]. If compared with semiconductor materials, polymer WGs are much more easily fabricated, since both lithographic and imprint technologies can be used without the need of any dry etching step.

**Figure 1.**
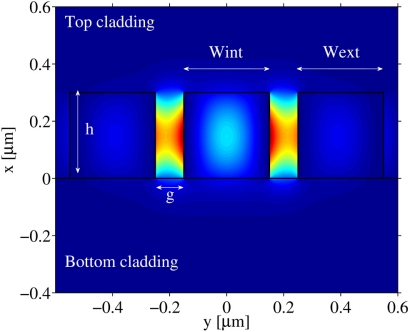
Cross section of a double slot waveguide, where *g* is the slot width, *h* is the slot height, and *Wint* and *Wext* are the internal and external wall width, respectively. Dielectric boundaries are depicted by black lines, while colour is proportional to the field intensity of quasi-TE fundamental mode, clearly showing the areas where the field is maximized.

The vertical slot configuration is considered in this paper. The cross section of a double slot WG is sketched in Figure 1 together with its relevant geometrical parameters. Simulations have been carried out using two different commercial packages, *i.e.*, a fully vectorial mode finder [10] and a full-vectorial FEM approach, largely used for photonic structures [11]. Then, a qualitative comparison has been made with a third simulation method based on an open source code [12]. We have considered both fundamental and higher order quasi-TE modes, but the fundamental quasi-TE mode is seen as a design target because the majority of the simulated high sensitivity SWGs are monomodal and the contribution of higher order modes, although not negligible, is weighted by their low modal indices. Thus, higher order modes would hardly contribute to the enhancement of device performance, especially because of their expected strong scattering losses.

## 3. Results and Discussion

### 3.1. Homogeneous Sensitivity of Slot Waveguides

**Figure 2.**
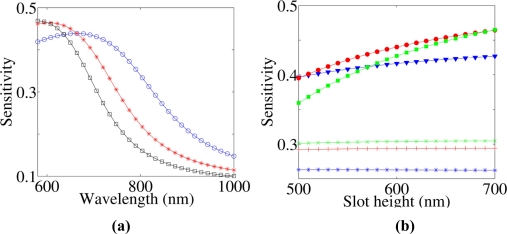
**(a)** Dispersion of ***S*** *versus* wavelength for a single SWG, 700 nm high and 200 nm wide, for three different slot widths: 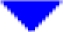 50 nm, 
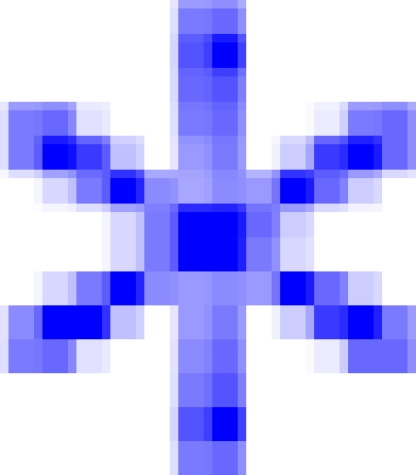
 80 nm and □ 100 nm. **(b)** ***S*** *versus* slot height at λ = 600 nm. Filled symbols ( 
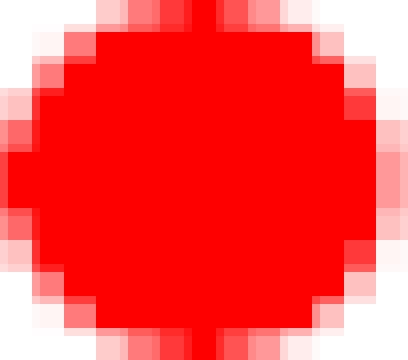
, 
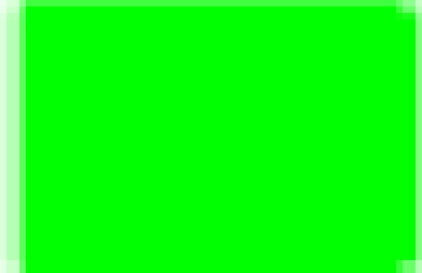
, 
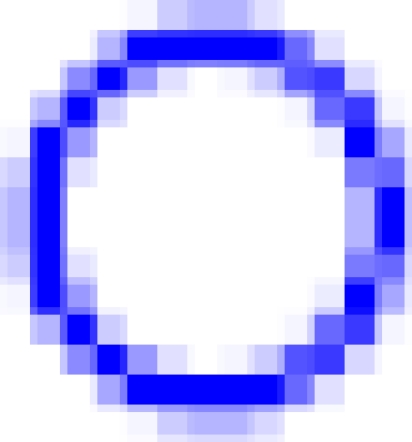
) refer to SWG having 200 nm wall widths, while crosses ( 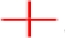, 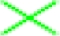, 
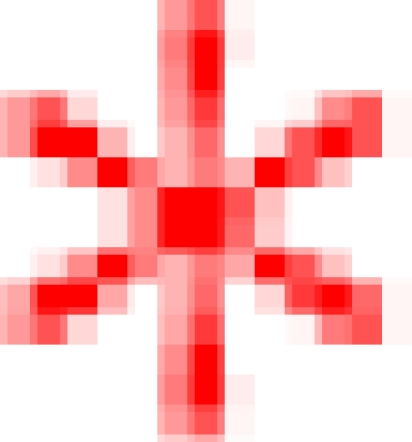
) to SWG with 300 nm wall width. For each set of data, three different slot widths are considered: 50 nm ( 
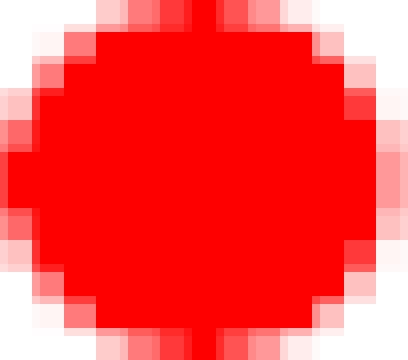
 and 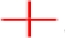), 80 ( 
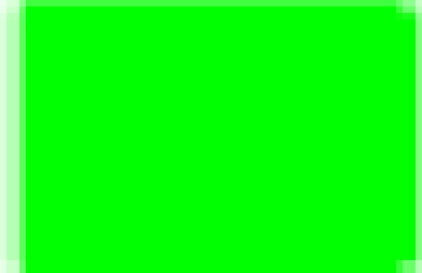
 and 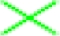) and 100 nm ( 
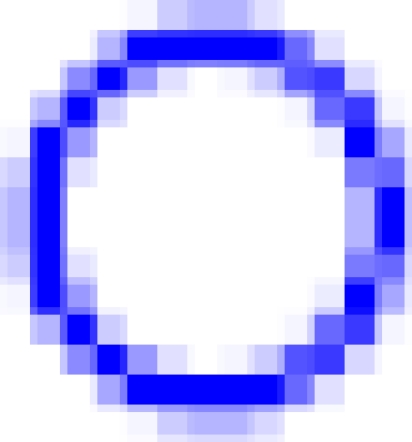
 and 
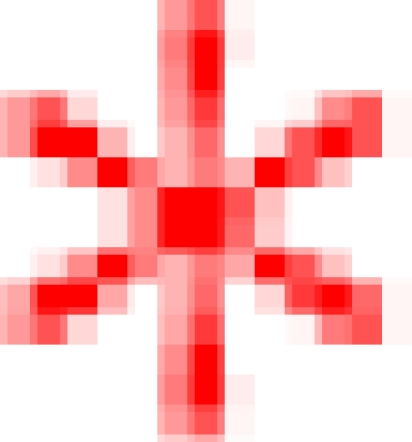
).

A common definition of WG sensitivity correlates the refractive index change directly to the field confinement [13] as:

(1) $$S=\frac{\partial n_{\mathrm{eff}}}{\partial n_{C}}=\frac{2n_{C}^{0}}{\eta_{0}P}{\iint_{R}\left|E\left(x,y\right)\right|}^{2}\mathrm{dxdy}$$

where $n_{C}^{0}$ is the unperturbed top cladding refractive index, *P* is the Poynting vector calculated over the entire simulation domain, *η_0_* is the free space impedance, *E* is the electric field vector and *R* is the integration domain (unless differently specified, the integral has been calculated over both the top cladding layer and the slot region). Equation (1) represents the definition of homogeneous sensitivity used in this work. Figure 2 shows some results of homogeneous sensitivity for a single LI SWG. Figure 2(a) reports the behaviour of ***S*** *versus* wavelength considering a single SWG, 700 nm thick and 200 nm wide walls. Three different slot widths are considered: 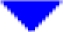 50 nm, 
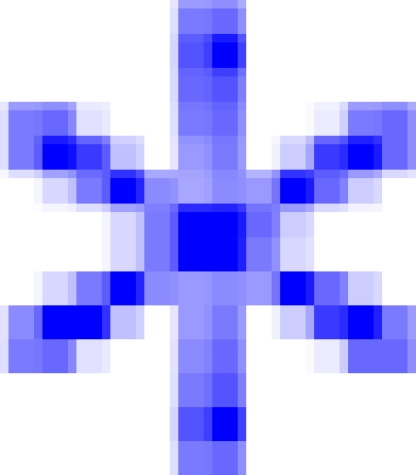
 80 nm and □ 100 nm. For each SWG geometry, the maximum ***S*** is achieved at the optimum trade-off between optical confinement in the slot (that is optimized at shorter wavelengths) and extension of evanescent field in the cladding (maximum at longer wavelengths). The same trend is observed about the dependence of ***S*** on the slot width. In fact, SWG with narrowest slot has the best confinement (guiding) properties while ***S*** is maximized at longer wavelengths. On the other hand, the largest slot produces the highest ***S*** at shorter wavelengths.

Figure 2(b) shows how ***S*** changes *versus* the slot height. Two sets of single LI SWGs, having different wall widths, have been considered, namely 200 nm (filled symbols) and 300 nm (crosses). For each set three different slots widths have been investigated: 50 nm ( 
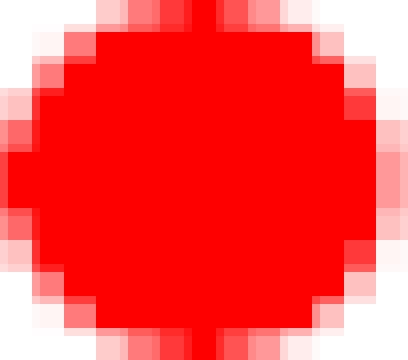
 and 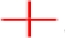), 80 ( 
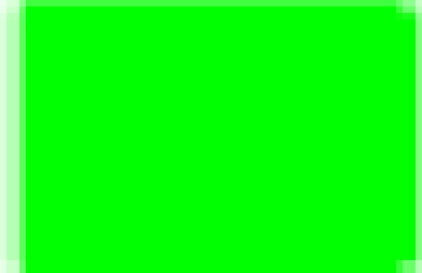
 and 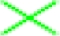) and 100 nm ( 
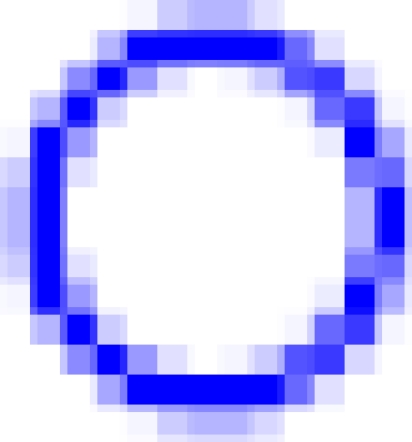
 and 
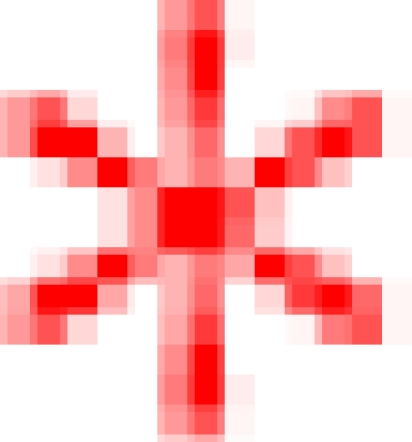
). In narrow SWGs a clear proportionality between ***S*** and slot height occurs. We have found that 80 nm wide slot allows to maximize ***S*** over the considered range of WG heights.

**Figure 3.**
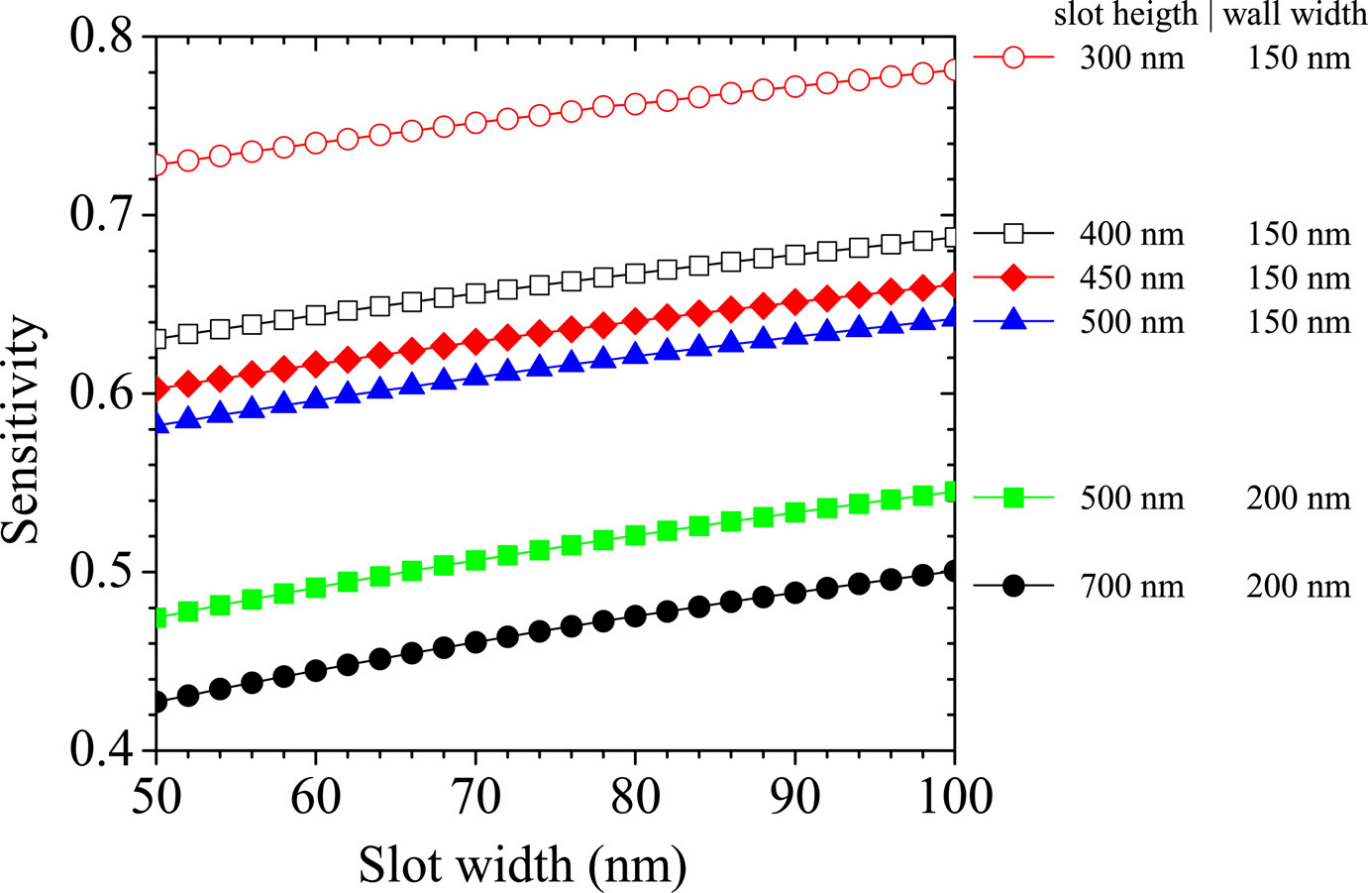
Sensitivity of a single LI SWG immersed in water (λ = 600 nm). It shows an inverse proportionality of ***S*** *versus* slot height, as expected. The thinner SWG shows ***S*** similar to that obtained with HI materials. The geometries considered are described in the legend.

Nevertheless, crossings of the three datasets indicate how the maximum ***S*** significantly depends on the actual SWG geometry, thus a general rule to design the optimal guiding structure for sensing is difficult to be directly derived. On the other hand, sensitivity of wider SWGs very slightly depends on the slot height (less than 1% change over the whole range). This fact suggests that wider walls make the WG to behave similarly to a classical rib structure, reducing the fraction of the optical mode that is confined into the slot region and fixing a lower limit to ***S*** for LI SWGs. The circumstance that a SWG careful design is fundamental in order to optimize ***S*** for a given SWG geometry is demonstrated by Figure 3, too. Here the top cladding and slot regions are assumed as filled with water (*n* = 1.33). We can note as ***S*** is greatly enhanced with respect to Figure 2, because the smaller refractive index contrast between wall materials and cladding, induces a greater mode expansion. The maximum ***S*** is achieved for the thinnest WG. Figure 3 reports changes in ***S*** as a function of slot width for SWG heights ranging from 450 nm to 700 nm. In these cases, ***S*** is inversely proportional to the slot height. The two open dot datasets report the values of ***S*** achievable in LI systems by reducing SWG heights to the minimum value allowed for guiding light. Clearly, the SWG effective indices with large ***S*** tend to be similar to the cladding refractive index. These calculations show that values of ***S*** similar to that of HI systems [14] are achievable in LI single slot structures although their index contrast is poorer and, similarly important, that ***S*** shows a weak dependence on the mode effective index, *i.e.*, better tolerance against fabrication defects.

### 3.2. Comparison between High and Low Refractive Index SWG

**Figure 4.**
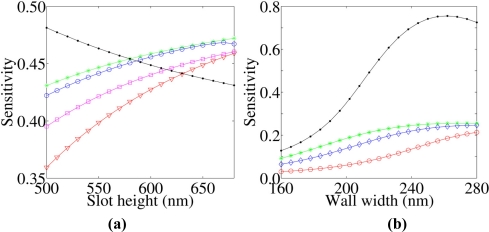
Comparison of ***S*** in HI (refractive index of 3.5) and LI (refractive index of 1.5) SWG. **(a)** ***S*** *versus* slot height. 
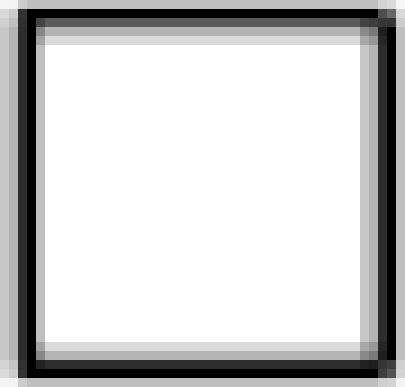
 are the data for a HI SWG, considering a working wavelength of 1,550 nm. The other data indicate LI SWG working at λ = 600 nm, with different number of slots (100 nm wide if not specified differently): 
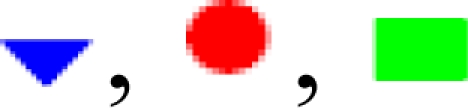
 single slot, 
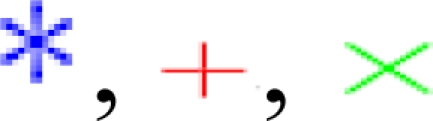
 single slot 80 nm wide, 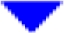 double slot, 
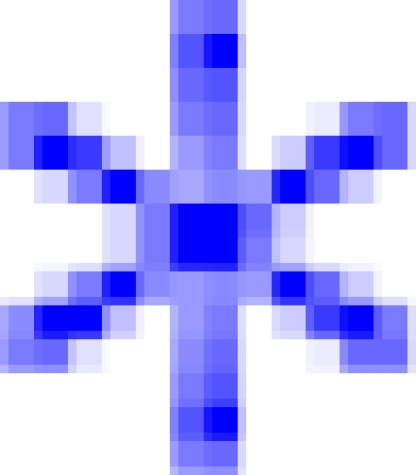
 triple slot. **(b)** ***S*** *versus* wall width. All SWGs are 250 nm thick, with slots 100 nm wide. 
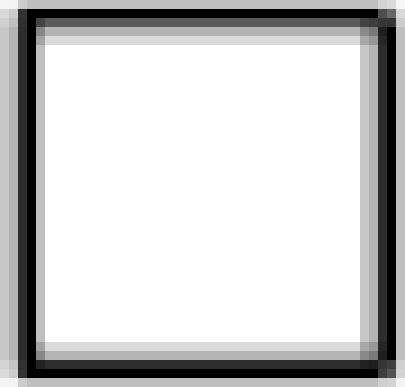
 are the data for a HI SWG at 1,550 nm, 
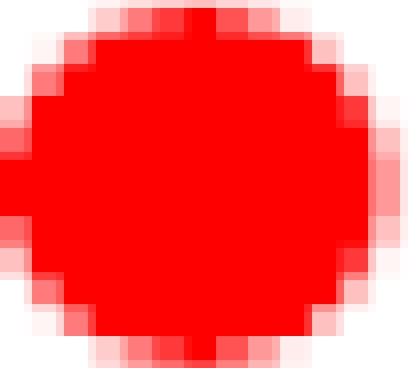
 single LI SWG, 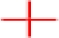 double LI SWG, 
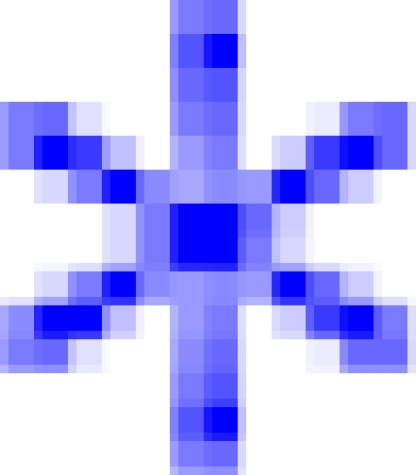
 triple LI SWG.

It is well known that the field enhancement inside the slot volume is proportional to the ratio of the material dielectric constants. In fact, field discontinuity at the interface scales as (*n_w_/n_g_*)^2^, with *n_w_* the SWG wall refractive index and *n_g_* the slot region index [4]. Figure 4 reports a comparison of ***S*** in single and multiple SWGs formed with HI and LI materials. The two panels have different scales because they show the optimal geometries maximizing ***S*** in LI materials as in Figure 4(a) and HI materials in Figure 4(b), respectively. In thick SWGs (*h* > 600 nm), LI SWG sensitivity exceeds that of HI materials, because the weak confinement mode of LI systems enhances the ***S*** value. In HI samples, the SWG gets multimode for thicker structures and, thus, ***S*** decreases because of a better redistribution of the guided field inside the dielectric regions. Figure 4(b) reports a more propitious geometry for HI materials that is achieved with a slot height of 250 nm. In this case, LI SWG only supports weakly guided modes and, consequently, ***S*** values are strongly reduced. On the contrary, the monomodal condition of HI SWG maximizes ***S***. Then, a couple of important considerations can be done:

1. While the field discontinuity ratio between HI and LI materials assume a value of 5.4 (considering dielectrics having refractive indices of 3.5 and 1.5 as in silicon on insulator technology), ***S*** scales only by a factor lower than 2 for properly designed SWGs. This circumstance further confirms the interest for LI dielectrics in slot waveguides for sensing.
2. For SWG geometries maximizing the sensitivity, the increase of ***S*** due to multiple SWGs tends to be minimal (Figure 4(a)). Nevertheless, the use of multiple SWGs can substantially improve ***S*** in liquid core sensor devices, as discussed in the next section.

### 3.3. Sensitivity of Multiple Slot Devices

**Figure 5.**
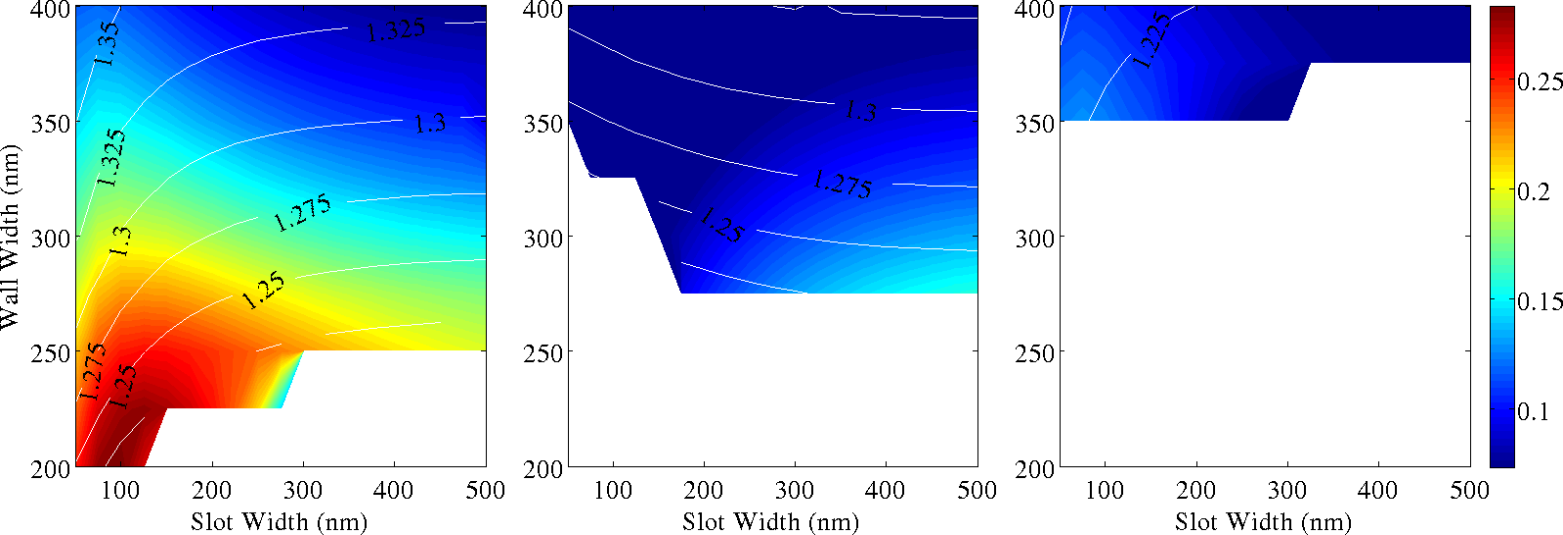
***S*** for single slot in case of **(a)** first, **(b)** second and **(c)** third mode (*h* = 700 nm, *Wext* = 200 nm). Superimposed to the 2D map there are a few contour lines reporting the mode effective index.

**Figure 6.**
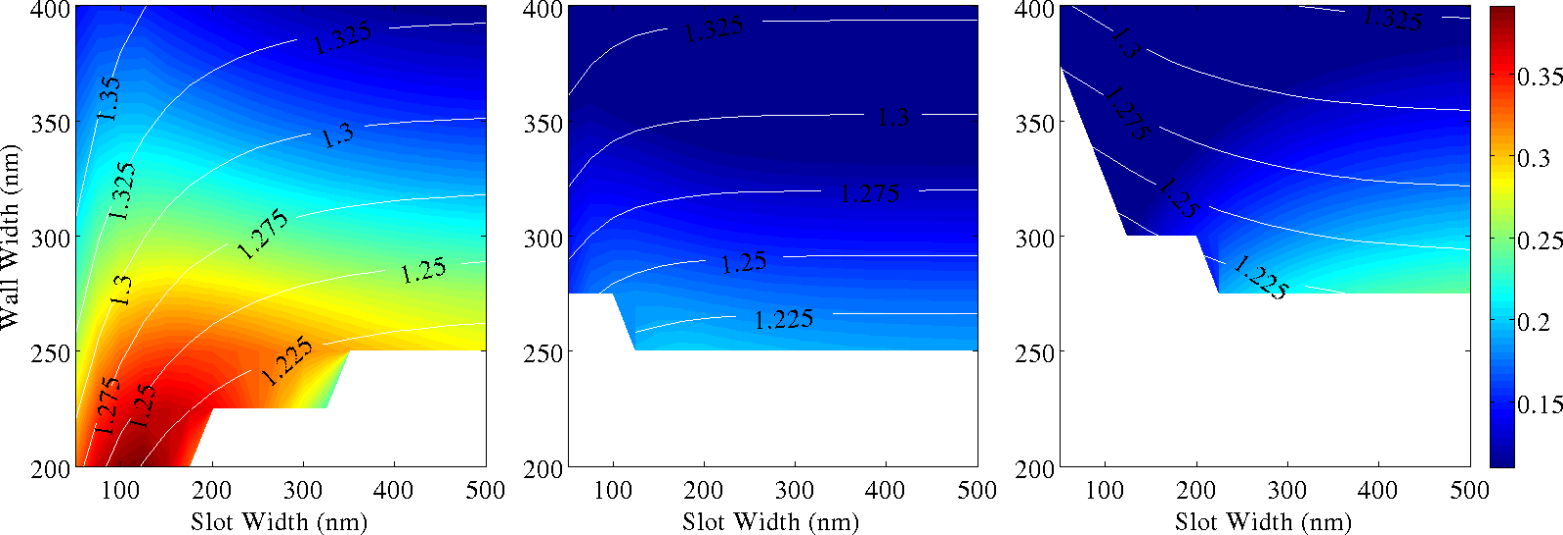
***S*** for double slot WG in case of **(a)** first, **(b)** second and **(c)** third mode (*h* = 700 nm, *Wext* = 200 nm). Superimposed to the 2D map there are a few contour lines reporting the mode effective index. Color bar limits range from ***S***_min_ = 0.02 to ***S***_max_ = 0.39 (λ = 600 nm).

**Figure 7.**
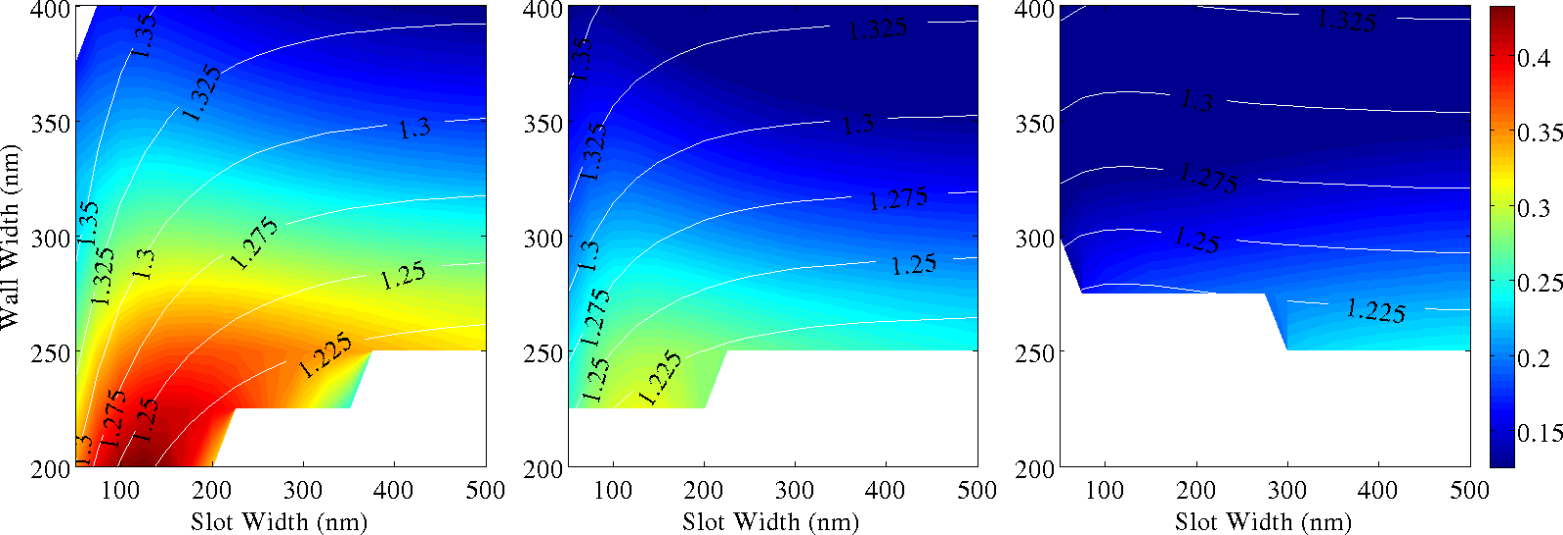
***S*** for triple slot WG in case of **(a)** first, **(b)** second and **(c)** third mode (*h* = 700 nm, *Wext* = 200 nm). Superimposed to the 2D map there are a few contour lines reporting the mode effective index. Color bar limits range from ***S***_min_ = 0.01 to ***S***_max_ = 0.45 (λ = 600 nm).

Figures 5–7 show 2D maps of ***S***, in single and multiple SWGs, *versus* both slot (g) and wall (Wint) widths. Here ***S*** is calculated integrating the field over the slot region only, to underline the role of the slot in sensing action. For all simulations, refractive indices of SWG, bottom and top cladding are 1.5, 1.2 and 1.0, respectively, and optical wavelength is again λ = 600 nm. These values correspond to a WG realized in polymethylmethacrylate (PMMA) deposited over a cladding layer of oxidized porous silicon [15]. A slot height *h* = 700 nm has been considered. In the next section it is shown that thicker WGs have greater sensitivity, but fabrication process becomes more and more complex for high aspect ratio structures.

Moreover the presence of higher order modes is characteristic of polymeric structures with such large heights, having different effective indices (shown in the figures as contour lines). A significant reduction of sensitivity is revealed in structures working with higher order modes, due to their poorer CF in the slot region. These graphs constitute fundamental guidelines for designing single mode, high sensitivity polymer SWG.

In double and triple SWG systems, the scan over wall widths *Wint* concerns only the internal dielectric walls, equivalent to change the inter-slot coupling effect, while the external ones are kept as fixed as *Wext* = 200 nm. Therefore, ***S*** is the sum of the contributions due to all structure slots. The white areas of Figures 5–7 refer to the cut-off regions, where guided modes cannot exist. As it is intuitive the use of multiple SWGs produces a better total electromagnetic field confinement inside the slots [9,16]. Single SWG is characterized by the most extended cut-off region. Due to the very low modal effective index, relatively high propagation losses should be expected in these structures. On the other hand, both double and triple SWGs have smaller cut-off regions, greater ***S*** values and slightly greater effective indices. Thus, they are potentially more robust waveguides. Scattering from the slot surfaces can have a detrimental role, nevertheless nearly ideal structures with extremely low roughness and vertical sidewalls were already demonstrated in polymeric materials [17,18] at submicron length scale, so that it is reasonable to assume very high quality of the samples realized in polymeric materials. In addition, it is of interest to compare the relative enhancement of ***S*** in SWG with a different number of slots. This parameter is defined as:

(2) $$S_{\mathrm{rel}}=\frac{S_{M}\;(s,W)}{S_{m}\;(s,W)}$$

where *M* and *m* refer to WG with greater and smaller number of slots, respectively, and (*s*, *W*) are the slot and wall widths.

Thus, *S_rel_* represents the ratio of the sensitivity between SWGs with different number of slots (3 *versus* 2, 2 *versus* 1 and 3 *versus* 1). By comparing these cases, it is observed that *S_rel_* grows proportionally to the effective index, but the trend is sublinear and negligible enhancement of ***S*** is expected in guided-wave structures with more than 3 slots. Our calculations show that the maximum *S_rel_* is of the order of 50% for double SWG and 70% for triple SWG (considering only the fundamental mode). Unfortunately, these large values are achieved in a region of low sensitivity (corresponding to large slots and wall widths) and reflect the stronger mode confinement achievable in WG with larger cross section.

Similar comments can be made for higher order modes. In these cases *S_rel_* increase up to an order of magnitude between multiple and single slots geometries, but only if very narrow slots are considered. In fact, for higher order modes, *S_rel_* decrease exponentially with the increase of the slot width. Thus smaller enhancements are found for the WG geometries maximizing ***S***, as shown in Figure 4. As it is clear from Figures 5–7, LI materials allow the fabrication of wide slots (up to 200 nm) without any strong reduction of CF. This is a great advantage if compared to HI materials. In fact, if applications for sensing liquids are considered, then larger slots will ease the liquid transport through the WG and the very narrow slots required by HI systems can be unworkable due to mass transport limitation on short timescales.

In conclusion, better behaviour in terms of sensitivity as well as larger range of available WG parameters (better fabrication tolerances) are achieved for SWG structures working with fundamental mode, with increasing the number of slots. However, these more complicated structures with respect to the single SWG are not always justified, although the sensing area is increased when a larger number of slots are included and they can still work with the first mode. Moreover, the reduction of sensitivity in SWGs working with second, third (or higher modes) is also clearly shown and does not allow a practical use of these propagation modes for sensing in many applications.

### 3.4. Applications of LI SWG

**Figure 8.**
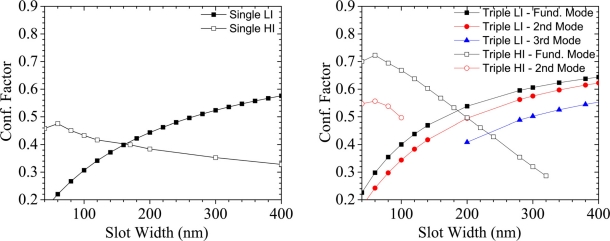
Dependence of CF on slot width in LI and HI single and triple SWG. Details of the simulated geometries are described in the legend. We consider λ = 00 nm and 1,550 nm for LI and HI materials, respectively.

LI SWG can be effectively used in evanescent fluorescent (or absorption) spectroscopy [19]. In these cases, the slot effect (discontinuity of the electric field at the dielectric boundaries) plays a secondary role and is not determinant for sensing properties, albeit it will enhance ***S***. Figure 8 reports the comparison between LI and HI SWG with the slot filled with liquid (without any loss of generality, we set a refractive index of 1.33 for the filling liquid, e.g., water). The exact dependence of CF on slot width is strongly dependent on the actual SWG geometry and a complete analysis should require a multi-parametric geometrical optimization that is out of the scope of this work. Nevertheless, a few geometries that roughly optimize this key parameter have been found and are here analyzed.

In Figure 8(a) the comparison between single SWGs realized in LI and HI materials is shown. As for Figure 5, λ = 600 nm for LI SWG and λ = 1,550 nm for HI SWG are assumed, respectively. The CF trend as a function of slot width is opposite for these two material systems. In this panel, we consider only the fundamental modes because second order modes appear on a few LI geometries, having a very low effective index not practical for sensing purposes. In HI SWG, wide slots can lower the CF, because the infiltrated liquid has a refractive index smaller than the bottom cladding, and thus the two SWG walls behave as a separated channel WG. On the contrary, in LI systems both mode effective index and CF grow as the slot becomes larger. HI SWG shows higher confinement in narrow slots but the highest value is achieved in LI SWG with wide slot, as expected. Figure 8(b) reports the comparison among triple SWGs. The general trends are similar to that for single SWG, but in this case we consider also higher order modes. HI multiple SWG is the configuration able to achieve the highest confinement value for the fundamental mode (that is supported up to 320 nm wide slot), while the second order mode exists only for narrow slot configurations (red, empty circles). On the contrary, the number of modes supported by LI systems increases with the slot width and so does the CF achievable. Because the refractive index of the bottom cladding is lower than that of the liquid, no cut-off conditions appear in LI SWG with wide slots. From the comparison between the two data panels, it can be noted as fundamental modes are much more enhanced in HI SWG, rather than in LI systems, with a relative improvement of the order of 50%. On the contrary, the multimodal behaviour of triple LI SWG allows the achievement of very high CF.

Thus, a slot volume, even filled with a liquid with refractive index higher than the WG walls, always enables an optimum overlap between the analyte (contained into the liquid) and the guided mode. Therefore, a stronger interaction is expected in a SWG compared with a fiber optic capillary (FOCap) or a standard rib WG. This occurs because the interaction is mediated by a truly guided mode, confined in the low index slot region, and not by low intensity evanescent mode tails. Moreover, in LI materials (e.g., polymers), a plethora of functionalization methods is available and the properties of these systems can be easily tailored to fulfil specific applications. We have focused this work only on the confinement properties of a straight SWG, and extensions to resonant structures are enough straightforward (as demonstrated in [20]). As we are going to show, in resonant systems LI SWGs can rival with FOCap sensor performances [21,22] and, thus, they can constitute a new platform for the development of integrated sensing devices. Absorption due to the evanescent field of an optical guided mode is described by the well known Lambert–Beer relation:

(3) $$A_{e\nu }=P_{e\nu }\gamma L$$

with *A_ev_* is the absorbance due to the evanescent field, *γ* is the absorption coefficient, *L* is the optical path length over which the absorption takes place and *P_ev_* is the optical power fraction in the evanescent tail of either a liquid core waveguide (LCW) or a FOCap. Considering a rough model assuming an homogeneous field distribution into a capillary section, *P_ev_* is given by [21,23]:

(4) $$P_{e\nu }=\frac{2\sqrt{2}}{3\pi }\frac{\lambda }{r_{\mathrm{core}}\mathrm{NA}}$$

where *r_core_* is the capillary wall thickness (or LCW core radius) and *NA* the numerical aperture. For common FOCap, *P_ev_* takes values of about 10^−3^ to 10^−2^. On the contrary, in LI SWG *P_ev_* reaches values of 0.3 (the power transmitted by the slot volume is defined by the *“filling factor”* and, depending on the SWG refractive index contrast, can be quite different from ***S*** value, as described in [24]). Moreover, to further increase the response of LI SWG, resonant structures can be used, such as microrings. Indeed, the effective optical length (*L_eff_*) in a microring is enhanced proportionally to its Q-factor [25]:

(5) $$L_{\mathrm{eff}}=\Gamma \frac{Q\lambda }{2\pi n_{\mathrm{eff}}}$$

with *Q* the cavity quality factor and Γ the optical mode CF. Using state-of-the-art experimental results for polymeric microrings, *Q* of the order of 2 × 10^4^ are easily achievable in 100 μm radius microrings [26,27], corresponding to *L_eff_* = 0.5 mm. On the other hand, the wavelength sensitivity of a SWG resonant microcavity can be written as:

(6) $$S_{w}=S\frac{\lambda }{n_{\mathrm{eff}}}$$

and it can assume values around 200 nm/RIU when *S* = 0.4, λ = 600 nm and *N_eff_* = 1.25. Then, the limit of detection (LOD) in these polymeric structures should be of the order of 10^−4^ RIU, if a standard wavelength resolution of 80 pm is assumed.

Thus, the absorption of a LI SWG single microring is comparable to that of a FOCap having length of a few of centimetres (the exact value depends on the mode confinement properties of the capillary). Moreover, it is important to note that the footprint of an LI SWG is of the order of 0.01 mm^2^, so that the required analyte volume can be of the order of a few of nanoliters. This value is orders of magnitude smaller than that of FOCap or LCW sensors [28]. Moreover, our integrated approach does not rely on mass transport. Spotting technologies could be used to selectively infiltrate the slot volume by capillary effect in an optofluidic integrated architecture.

## 4. Conclusions

In this article we discussed the use of very low refractive index materials, as suitable candidates to fabricate slot waveguides for sensing applications. We demonstrate that a high value of homogeneous sensitivity, comparable to that achievable in HI semiconductor material systems is compatible with LI materials. In particular, the sensitivity in LI material systems is shown as reduced by a factor much smaller than the ratio of refractive indices of the materials composing HI and LI photonic structures, respectively. This is particularly interesting for practical use since LI materials, such as polymers, are extremely flexible hosts that can be either surface functionalized (e.g., processed in order to give the necessary chemical selectivity to the sensor) or easily infiltrated (e.g., including active media inside, enabling the realization of complex WG arrays). From a microsystem point of view, the use of visible light based optical sensors, as the LI SWG described here, has a significant cost advantage with respect to NIR based optical sensor, as the silicon based HI SWG. Indeed, both sources and detectors are much cheaper in the visible than in NIR. Important considerations about the use of LI SWG as integrated sensor for evanescent absorption (or fluorescence) analysis were also discussed. The use of LI SWG instead of FOCap has a number of important advantages, mainly due to the better control of the field distribution in the slot volume and the consequent enhanced light-matter interaction. Among the most important benefits, we stress a strong interaction between the optical field and the analytes, a required analyte volume, some orders of magnitude smaller and the possibility to design integrated optical circuits enabling the development of complex WG sensor arrays and lab-on-a-chip photonic systems. Results presented here concentrate on very low index material systems but they can be straightforwardly extended to many other material platforms (e.g., Si_3_N_4_/SiO_2_). Although our results in this paper are theoretical, we are confident that these structures can be easily fabricated by well standardized polymer technologies for photonics.
